# Effect of multimodal CT angiography/perfusion sequencing on alteplase door-to-needle time

**DOI:** 10.1186/s12883-026-05105-y

**Published:** 2026-06-30

**Authors:** Zlatan Coralic, Benjamin Michaels, Kenneth Truong, Ralph Wang, S. Andrew Josephson, Anthony S. Kim

**Affiliations:** 1 Departments of Pharmacy and Emergency Medicine, University of California San Francisco, San Francisco, CA US; 2 Department of Pharmacy, University of California San Francisco, San Francisco, CA USA; 3 Department of Emergency Medicine, University of California San Francisco, San Francisco, CA USA; 4 Department of Neurology, Weill Institute for Neurosciences, University of California San Francisco, San Francisco, United States

**Keywords:** Acute ischemic stroke, Door-to-needle time, Stroke process, Multimodal imaging

## Abstract

**Background and purpose:**

Multimodal computed tomography angiography and perfusion (CTA/CTP) is increasingly used in the emergency department (ED) evaluation of acute ischemic stroke (AIS) but may delay thrombolysis. We examined whether the sequencing of multimodal CTA/CTP relative to thrombolytic administration affects door-to-needle (DTN) time.

**Methods:**

We conducted a retrospective cohort study of adult patients with AIS treated with intravenous alteplase at a tertiary, academically affiliated ED that is part of a certified Comprehensive Stroke Center between January 1, 2012, and August 1, 2023. All patients underwent an initial noncontrast CT (NCCT), followed by either a contrast-first workflow (i.e. CTA/CTP) or were given alteplase before proceeding to contrast administration. The primary outcome was DTN time, analyzed using univariate methods and multivariable quantile regression adjusting for stroke severity, pre-alteplase antihypertensive use, thrombectomy (as a correlate for large-vessel occlusion), primary language, and bedside ED pharmacist presence.

**Results:**

Among 1,382 AIS encounters, 167 patients met inclusion criteria; 148 underwent CTA/CTP before alteplase and 19 received alteplase before contrast administration. Median DTN time was significantly shorter in the alteplase-first group (20 minutes [interquartile range (IQR), 15–26]) compared with the CTA/CTP-first group (44 minutes [IQR, 32–56]; P<0.001). After adjustment, alteplase-first administration remained independently associated with faster DTN time (−25 minutes; 95% CI, −34 to −16). Higher stroke severity and ED pharmacist presence were also associated with shorter DTN, whereas pre-alteplase antihypertensives, primary language, and thrombectomy were not.

**Conclusions:**

A contrast-first strategy incorporating multimodal CTA/CTP before alteplase administration was associated with approximately twofold longer DTN times. These findings suggest that obtaining CTA/CTP before thrombolytic administration may substantially prolong DTN times. Larger studies are needed to confirm the magnitude of this association and to identify patient populations most likely to benefit from alternative imaging workflows.

## Introduction/background

Acute ischemic stroke (AIS) is a leading cause of cardiovascular related death and disability in the United States [1]. AIS constitutes 87% of all strokes and may lead to permanent brain injury within minutes.3,4 Rapid identification of patients with stroke, transport, and treatment with thrombolytics has been shown to improve functional outcomes and decrease neuronal loss [1–4].

Patients suspected of having a stroke are commonly evaluated in the emergency department (ED). After initial stabilization, rapid non-contrast enhanced computed tomography (NCCT) imaging of the brain is performed to rule out hemorrhage and to potentially show early signs of infarction [1]. Current guidelines, based on moderate-quality non-randomized studies (Class I, Level of Evidence: B-non randomized) strongly recommend that thrombolytics should be given to eligible patients based on the NCCT alone. These guidelines stress that fibrinolytic therapy should not be delayed for advanced multimodal imaging, such as CT-angiography (CTA) and CT-perfusion (CTP), unless there is substantial diagnostic uncertainty [1].

In the ED, several predictors have been associated with delays in thrombolytic administration, including delays in pre-hospital transport, history gathering and clinical examination, establishing intravenous (IV) access, consulting specialty services, and addressing concurrent acute conditions (e.g., hypertension, respiratory distress) [5–8]. Furthermore, obtaining imaging studies that require IV contrast administration has also been shown to be associated with delays in receiving thrombolysis [6, 7, 9]. While these contrast studies offer detailed insights into cerebral hemodynamics and ischemic areas, the original trials of thrombolytics were based on NCCT alone, and deferring thrombolytic administration until after the additional acquisition and interpretation time for multimodal CT scans may result in delays up to 20 min or more, potentially doubling door-to-needle (DTN) times [9]. Iglesias et al. [6] reported a 13.4% increase in DTN times due to imaging studies that require IV contrast administration and Choi et al. [7] found that DTN times increased by an average of 17 min when advanced imaging was obtained. Given that during AIS, each passing minute results in the death of millions of neurons, such delays represent a critical clinical concern.

Over the past decade, we have observed variations in the sequencing of alteplase and contrast administration for multimodal CT during Code Stroke evaluations. Here we describe the timing of multimodal CT relative to alteplase administration and its impact on DTN times.

## Methods

### Study design and setting

This was a retrospective analysis of electronic health record data of patients evaluated at an urban tertiary care academic ED between January 1, 2012 and August 1, 2023. The volume of ED encounters is approximately 45,000 per year and the center is certified as a Comprehensive Stroke Center with academic training programs in emergency medicine, radiology, neuroradiology, neurology, vascular neurology, neurosurgery, and vascular neurosurgery, [and pharmacy and nursing] and has 24/7 in-person neurology coverage.

A multidisciplinary Code Stroke team evaluates and treats all patients suspected of stroke presenting within 24 h of symptom onset. The team is led by a neurology resident in collaboration with an emergency medicine resident and an attending, nurses, radiologists, and an emergency medicine clinical pharmacist. The pharmacists are generally present at the bedside from 8 am to midnight and assist with contraindication screening and alteplase administration. Incoming patients are rapidly triaged, evaluated, and promptly taken for a stroke protocol CT which includes a NCCT and advanced neuroimaging with CTA and CTP. The team leader obtains a detailed history from patient and family, obtains a National Institute of Health Stroke Scale (NIHSS) score, [10] and issues medication orders (e.g., anti-hypertensives, thrombolytics). Diagnostic and treatment decisions are coordinated between the lead neurology resident, the neurovascular fellow and attending, and the radiology resident and neuroradiology fellow.

### Study participants and intervention studied

We identified all adult patients (≥ 18 years old) diagnosed with AIS at hospital discharge who were given alteplase in the ED. Alteplase was the only thrombolytic that was in use for AIS during the study period. Patients were dichotomized based on sequencing of the contrast imaging study with alteplase administration post NCCT (i.e., contrast first vs. alteplase first). We excluded patients who received alteplase but had no contrast studies after the NCCT in the ED and patients whose records were incomplete or unavailable .

### Data collection

Data points of interest were extracted from the electronic health record reporting server (Epic Clarity) using Structured Query Language commands. The extraction code was developed, tested, and revised during a pilot phase. Any extreme or nonsensical values identified were checked for accuracy against a manual review of the chart in order to adjust the pilot code prior to extraction of the final analysis dataset. Additionally, variables related to the timing and sequencing of the contrast studies and alteplase were manually confirmed via chart review for all patients in the final cohort.

### Primary measure of interest

The primary measure of interest was door-to-needle (DTN) time. This was calculated by subtracting the ED triage time (door time) from the time of alteplase administration (needle time). The timing of contrast administration for the multimodal CT was based on the timestamp on the initial image acquired during the CT-angiogram timing series, specifically the first image at the heart level that visibly demonstrated the arrival of IV contrast. Additionally, we abstracted predictors that could have contributed to delays in DTN times based on previously published reports, including [5, 6, 8, 9, 11] presenting NIHSS, the need to administer IV anti-hypertensives, the presence of IV access upon ED arrival (defined as an IV line started in the pre-hospital setting), known contrast allergy, the patient’s primary language, subsequent thrombectomy, and bedside presence of an ED pharmacist.

### Statistical analysis

We describe continuous data using medians and interquartile ranges, and categorical data using proportions, analyzing differences using Mann-Whitney U test and Chi-squared test, respectively. Kaplan-Meier failure function curves were used to visualize time-to-event data (i.e., DTN times). Because DTN times are inherently right-skewed, we employed quantile regression to estimate median time differences between groups while adjusting for NIHSS category, presence of an established IV line at ED arrival, receipt of IV antihypertensives prior to alteplase, primary language (English vs. non-English), presence of an ED pharmacist at the bedside, and the need for subsequent thrombectomy. All analyses were performed using Stata version 18.0 (StataCorp LLC, College Station, TX).

## Results

**Fig. 1:**
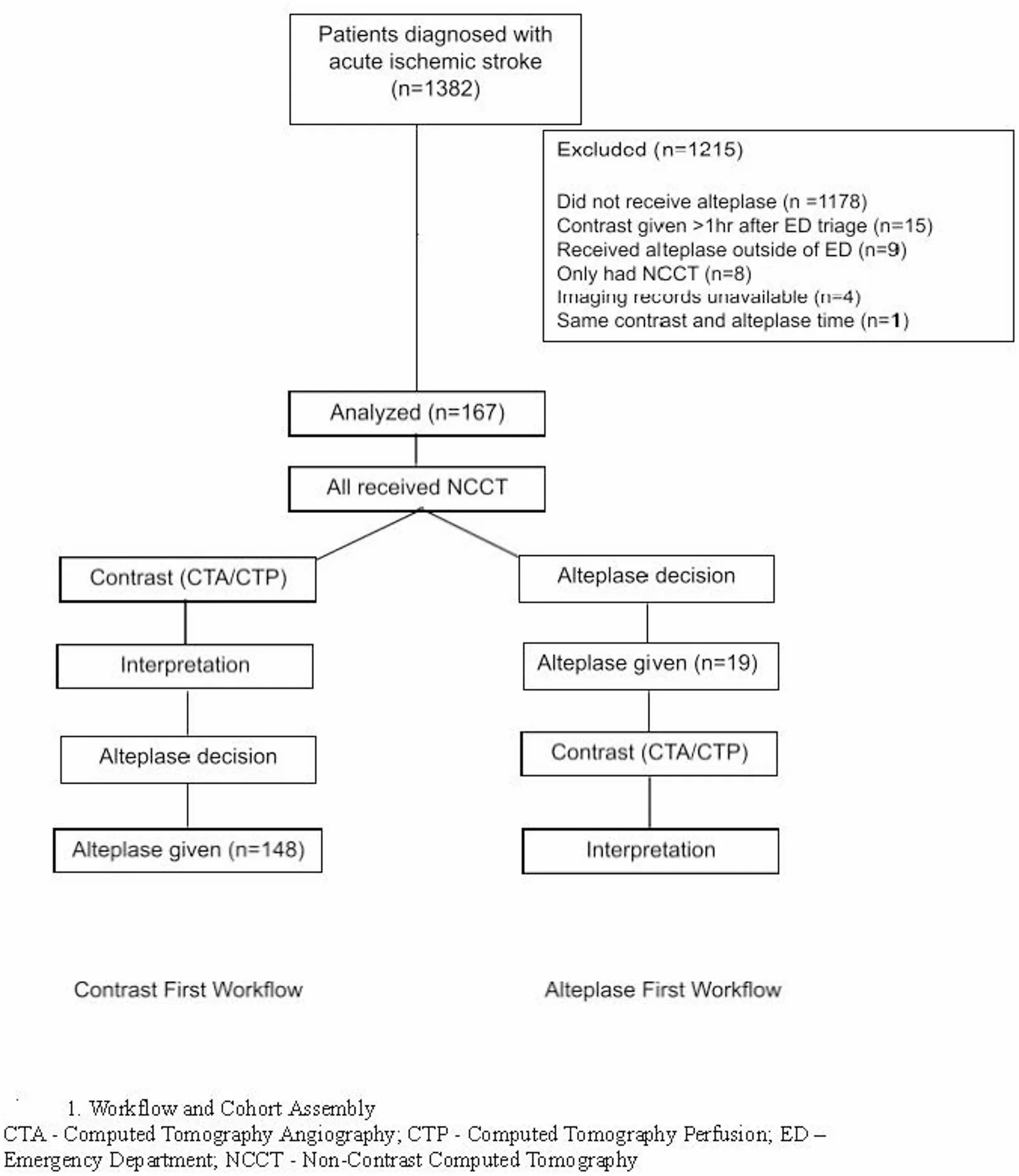
Cohort assembly. CTA - Computed Tomography Angiography; CTP - Computed Tomography Perfusion; ED -Emergency Department; NCCT - Non-Contrast Computed Tomography

A total of 1,382 adult patients were diagnosed with AIS with 167 (12.1%) receiving both alteplase and IV contrast in the ED and were included in the analysis; 148 received contrast first and 19 received alteplase first (Fig. 1). Patients given alteplase first were more often primarily English-speaking (84.2% vs. 61.6%, *p* = 0.05). No other baseline differences were observed between groups (Table 1).

**Table 1:** Patient demographics, triage, and disposition

	First intervention		
	Contrast	Alteplase	*P*
N	148 (88.6%)	19 (11.4%)	
Age in years, median(IQR)	78 (67–89)	81 (70–88)	0.731
Female sex	86 (58.1%)	10 (52.6%)	0.832
Weight in kg, median (IQR)	70 (58–82)	78 (64–86)	0.403
Race			
Asian	59 (39.9%)	5 (26.3%)	0.332
Black/African American	11 (7.4%)	2 (10.5%)	
Latinx	9 (6.1%)	2 (10.5%)	
White	54 (36.5%)	10 (52.6%)	
Other/Unknown	15 (10.1%)	0 (0.0%)	
English primary language	90 (60.8%)	16 (84.2%)	0.046
Mode of arrival			
Ambulance	128 (86.5%)	19 (100.0%)	0.233
Car/Taxi	13 (8.8%)	0 (0.0%)	
Walk-in/Other	7 (4.7%)	0 (0.0%)	
Triage acuity			
1	27 (18.5%)	6 (33.3%)	0.292
2	116 (79.5%)	12 (66.7%)	
3	3 (2.1%)	0 (0.0%)	
Discharge Disposition			
Rehabilitation/SNF/LTC	93 (63.3%)	13 (68.4%)	0.870
Deceased	13 (8.8%)	1 (5.3%)	
Home	38 (25.9%)	5 (26.3%)	
Other	3 (2.0%)	0 (0.0%)	

*IQR* Interquartile range, *kg* kilograms, *LTC* Long term care facility, *SNF* Skilled nursing facility

**Fig. 2:**
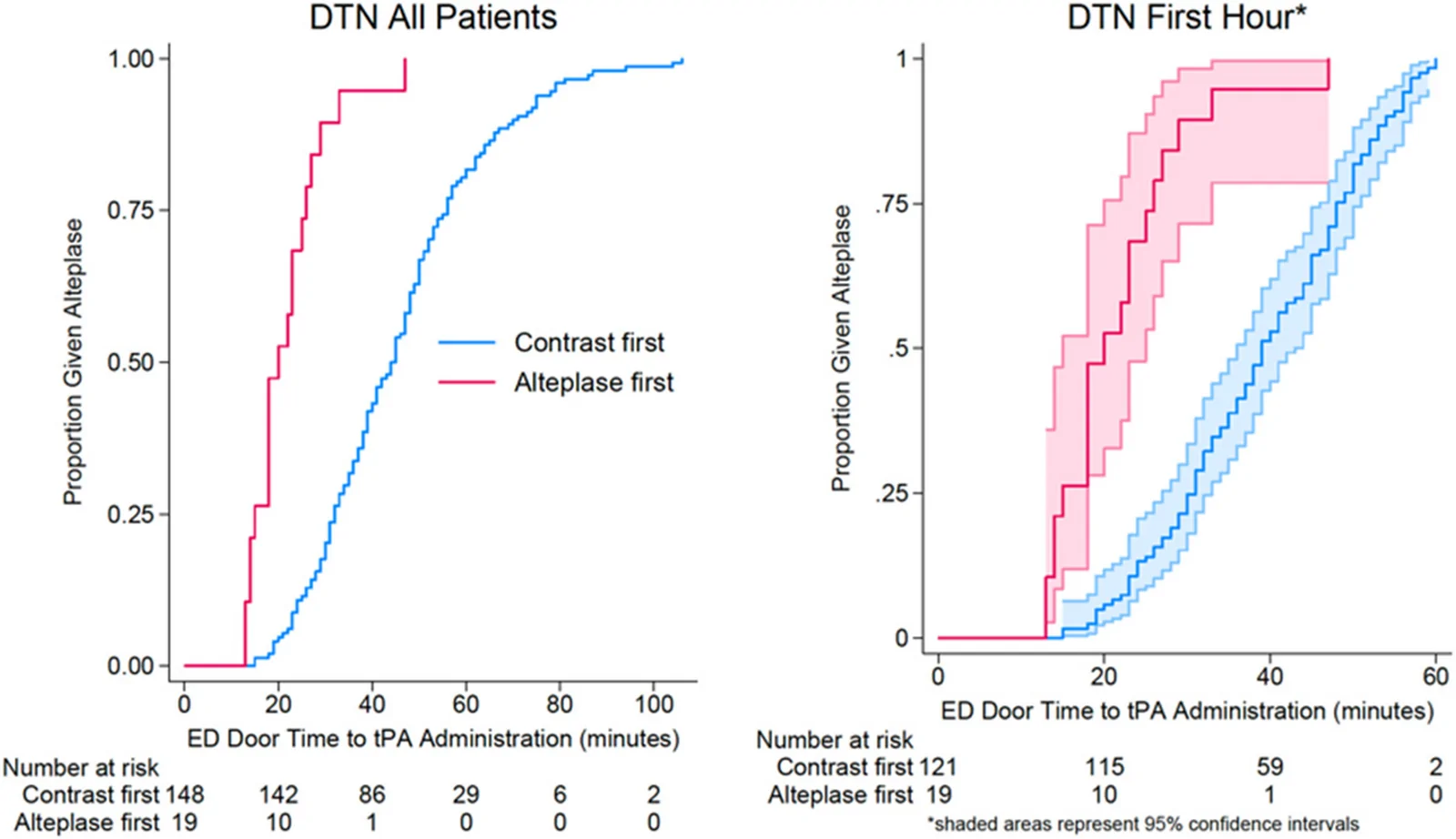
Kaplan-Meier curves for door-to-needle times in all patients and within the first hour

Overall, DTN was achieved for 140/167 (83.8%) within 60 min, 98/167 (58.7%) within 45 min, and 47/167 (28.1%) within 30 min. Patients receiving alteplase first had shorter DTN times compared to patients receiving contrast first (20 min [IQR 15–26] vs. 44 min [IQR 32–56], *p* < 0.001). Time from alteplase to contrast in this group was also shorter than the contrast-to-alteplase interval among patients given contrast first (7 min [IQR 5–9] vs. 26 min [IQR 18–36], *p* < 0.001). In addition, alteplase-first patients were more likely to have prehospital IV access (47.4% vs. 18.5%, *p* = 0.004). Additional measures of interest are summarized in (Table 2; Fig. 2).

**Table 2:** Measures of interest

	First intervention		
	Contrast	Alteplase	*P*
N	148 (88.6%)	19 (11.4%)	
Presenting NIHSS	12 (5–19)	11 (6–17)	0.578
NIHSS category			
Minor, 0–4	39 (26.4%)	4 (21.1%)	0.485
Moderate, 5–15	51 (34.5%)	10 (52.6%)	
Moderate/Severe, 16–20	34 (23.0%)	3 (15.8%)	
Severe, 21+	24 (16.2%)	2 (10.5%)	
Door to alteplase in min, median (IQR)	44 (32–56)	20 (15–26)	< 0.001
Contrast to alteplase/alteplase to contrast time in min, median (IQR)	25 (18–36)	7 (5–9)	< 0.001
Door time to contrast in min, median(IQR)	17 (12–22)	28 (21–42)	< 0.001
Thrombectomy	30 (20.3%)	2 (10.5%)	0.310
Pre-alteplase SBP > = 185	30 (20.3%)	4 (21.1%)	0.143
Pre-alteplase DBP > = 110	27 (18.2%)	2 (10.5%)	0.139
IV Anti-HTN One Hour Prior to Alteplase	21 (14.2%)	1 (5.3%)	0.279
IV Started Prehospital	27 (18.2%)	9 (47.4%)	0.004
Allergy to contrast	2 (1.4%)	1 (5.3%)	0.227
ED Pharmacist Bedside	135 (91.2%)	18 (94.7%)	0.602

*ED* Emergency department, *DBP* Diastolic blood pressure, *NIHSS* National Institutes of Health Stroke Scale, *SBP* Systolic blood pressure

**Fig. 3:**
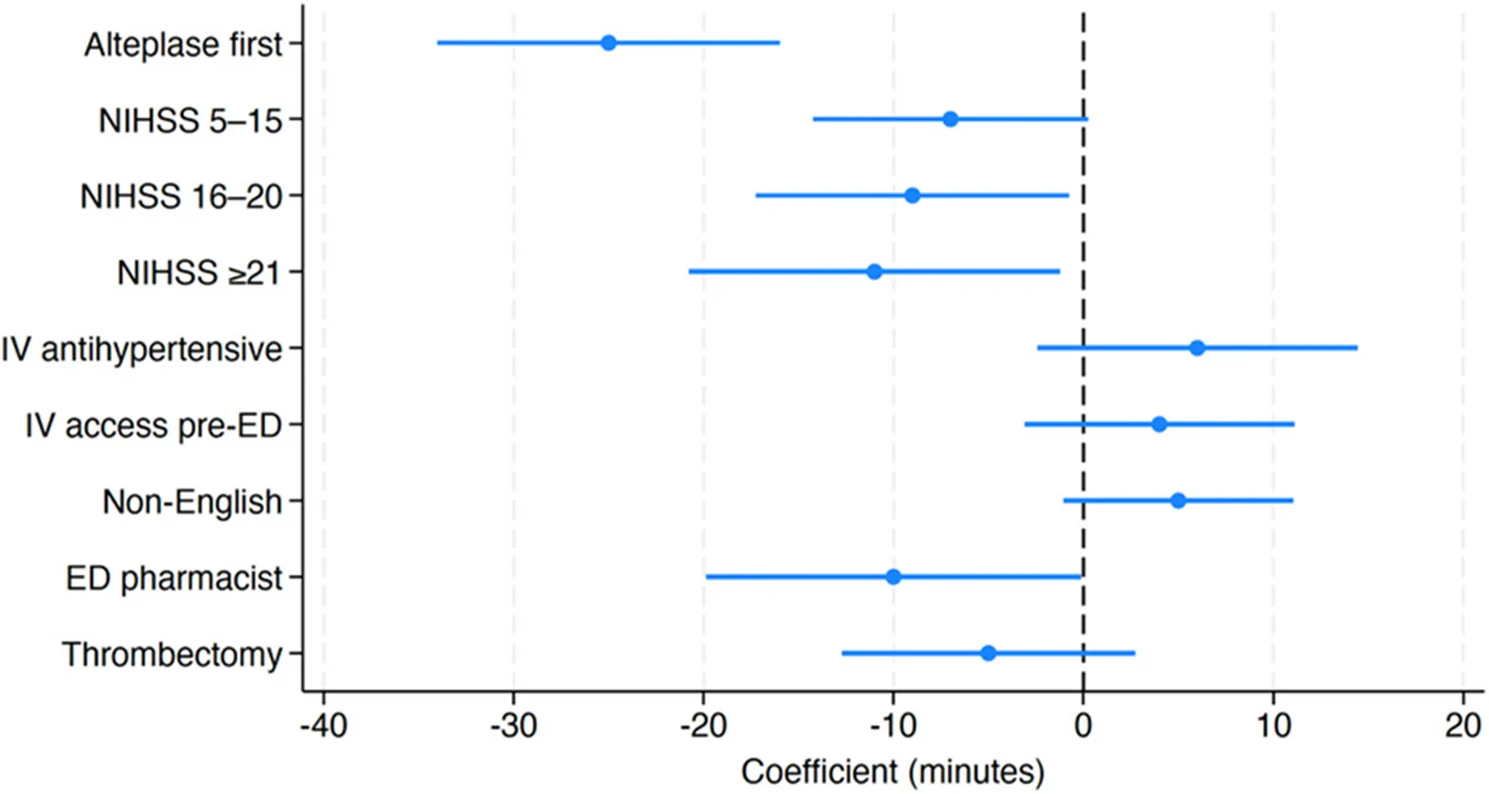
Multivariable quantile regression forest plot for selected predictors

In multivariable analysis, administering alteplase before contrast was independently associated with shorter median DTN time (− 25 min [95% CI − 34 to − 16]). Increasing NIHSS category was likewise associated with faster treatment. The presence of an ED pharmacist during code stroke response was also associated with reduced DTN (− 10 min [95% CI − 20 to − 0.1]). DTN times were not significantly associated with subsequent mechanical thrombectomy, pre-alteplase IV antihypertensive use, or primary language (Fig. 3; Table 3 [data]).

**Table 3:** Multivariable quantile regression output

Variable	Coefficient (min)	95% CI	*P* value
Alteplase first	-25	(-34.0, -16.0)	<0.001
NIHSS 5-15	-7	(-14.2, 0.2)	0.057
NIHSS 16-20	-9	(-17.2, -0.8)	0.032
NIHSS ≥21	-11	(-20.8,-1.2)	0.027
IV antihypertensive	6	(-2.4, 14.4)	0.161
IV pre-ED	4	(-3.1, 11.1)	0.267
Non-English language	5	(-1.0, 11.0)	0.104
ED Pharmacist	-10	(-19.9, -0.1)	0.047
Thrombectomy	-5	(-12.7, 2.7)	0.202

## Discussion

Our study demonstrates the association between the sequencing of multimodal CT and alteplase administration in patients with AIS, with administering alteplase before contrast correlating with faster DTN times. The observed 24-minute median reduction in DTN time for patients receiving alteplase first may translate into improved outcomes given the time-sensitive nature of ischemic stroke treatment. These findings are consistent with current guidelines [1] that advocate for thrombolysis based on NCCT alone unless there is substantial diagnostic uncertainty, and highlight the potential delays introduced by incorporating multimodal CT into the acute workflow before alteplase administration.

The underlying mechanisms driving this association are likely multifactorial. While multimodal CT provides valuable information regarding penumbral core and collateral circulation and can help establish a vascular etiology to a patient’s presentation, acquisition and interpretation of these imaging studies necessitate additional time, as evidenced by the delays reported in prior literature and corroborated by our findings. Our data suggest that waiting for the complete multimodal CT results adds approximately 25 min to the DTN time, potentially exceeding the benefits gained from the additional information provided in some cases. This is especially true in patients who are clearly eligible for thrombolysis based on clinical presentation and NCCT alone, where delaying treatment for confirmatory multimodal imaging may outweigh its value. Future investigations should evaluate workflow factors contributing to post-imaging treatment delays.

Interestingly, we observed that patients receiving alteplase first were more likely to have pre-hospital IV access and known contrast allergies. The presence of established IV access undoubtedly facilitated faster medication administration, while the awareness of a contrast allergy likely prompted clinicians to prioritize thrombolysis over further contrast-enhanced imaging. This underscores the importance of incorporating pre-hospital data and patient history into the acute stroke workflow.

Our findings are consistent with prior studies demonstrating the impact of various factors on DTN times. For example, the association between pre-hospital IV access and faster DTN times aligns with research highlighting the benefits of an optimized system of pre-hospital care [1]. Furthermore, the positive association between the presence of an ED pharmacist and faster DTN times reinforces the value of multidisciplinary care in stroke management. Recent evidence from a systematic review and meta-analysis demonstrated that emergency medicine pharmacist involvement was associated with a 14.6-minute reduction in DTN times and increased achievement of guideline-recommended treatment benchmarks, supporting the role of pharmacists as integral members of stroke response teams [12]. At our institution, ED pharmacists participate in the majority of code stroke activations, assisting with inclusion and exclusion screening, identification of contraindications, and the preparation and administration of thrombolytic and supportive therapies, providing medication-specific expertise that may help streamline treatment workflows and reduce delays.

This study has several limitations. Its retrospective, single-center design limits generalizability and introduces potential selection bias, especially in the setting of unbalanced groups. Although we adjusted for measured confounders, treatment sequence was not randomized, and residual confounding and confounding by indication is likely. Because fewer patients underwent an alteplase-first workflow, the adjusted analyses should be viewed as exploratory and hypothesis-generating rather than definitive estimates of effect. Clinicians may have preferentially selected an alteplase-first approach for patients with more obvious or severe stroke presentations, while contrast-first imaging may have often been used in diagnostically uncertain cases, including stroke mimics or seizure, which would be expected to delay thrombolysis. Ultimately, our study design is unable to distinguish the effect of imaging sequence from the effect of case selection. Furthermore, we did not evaluate clinical outcomes such as symptomatic intracranial hemorrhage or functional status, limiting assessment of the clinical impact of faster DTN times. Time-of-day effects and secular trends in imaging and thrombolysis practices over the study period were not formally analyzed and may have contributed to observed associations, including the apparent pharmacist effect. Finally, as many systems transition to tenecteplase, it is unknown if its single-bolus administration may attenuate the influence of imaging sequence on DTN and warrants further study.

Despite these limitations, our study contributes hypothesis-generating insights into the relationship between multimodal CT imaging sequence and door-to-needle times in acute ischemic stroke. The observed association between obtaining CTA/CTP before alteplase administration and longer door-to-needle times highlights the importance of understanding how diagnostic workflows may influence treatment timeliness. Further studies incorporating clinical outcomes, detailed imaging characteristics, and multicenter populations are needed to better define the impact of imaging sequence on stroke care and patient outcomes.

## Data Availability

The data that support the findings of this study were extracted from the University of California, San Francisco (UCSF) Medical Center electronic health record and contain protected health information (PHI). In accordance with institutional policies, HIPAA regulations, and the conditions of Institutional Review Board approval, these data are not publicly available. De-identified data may be made available from the corresponding author upon reasonable request, subject to review and approval by UCSF and any applicable regulatory or institutional requirements.

## Abbreviations

AIS: Acute Ischemic Stroke
CTA: Computed Tomography Angiography
CTP: Computed Tomography Perfusion
DBP: Diastolic Blood Pressure
DTN: Door–to–Needle
ED: Emergency Department
HTN: Hypertension
IQR: Interquartile Range
IV: Intravenous
kg: Kilogram
LTC: Long–Term Care Facility
NCCT: Noncontrast Computed Tomography
NIHSS: National Institutes of Health Stroke Scale
SBP: Systolic Blood Pressure
SNF: Skilled Nursing Facility
